# Extracellular-vesicles delivered tumor-specific sequential nanocatalysts can be used for MRI-informed nanocatalytic Therapy of hepatocellular carcinoma

**DOI:** 10.7150/thno.46124

**Published:** 2021-01-01

**Authors:** Han Wu, Hao Xing, Meng-Chao Wu, Feng Shen, Yu Chen, Tian Yang

**Affiliations:** 1 Department of Hepatobiliary Surgery, Eastern Hepatobiliary Surgery Hospital, Second Military Medical University, Shanghai 200438, P. R. China.; 2 School of Life Sciences, Shanghai University, Shanghai 200444, P. R. China.

**Keywords:** extracellular vesicles, catalytic nanomedicine, theranostics, hepatocellular carcinoma

## Abstract

**Background:** Conventional therapeutic strategies for advanced hepatocellular carcinoma (HCC) remains a great challenge, therefore the alternative therapeutic modality for specific and efficient HCC suppression is urgently needed.

**Methods:** In this work, HCC-derived extracellular vesicles (EVs) were applied as surface nanocarrier for sequential nanocatalysts GOD-ESIONs@EVs (GE@EVs) of tumor-specific and cascade nanocatalytic therapy against HCC. By enhancing the intracellular endocytosis through arginine-glycine-aspartic acid (RGD)-targeting effect and membrane fusion, sequential nanocatalysts led to more efficient treatment in the HCC tumor region in a shorter period of time.

**Results:** Through glucose consumption as catalyzed by the loaded glucose oxidase (GOD) to overproduce hydrogen peroxide (H_2_O_2_), highly toxic hydroxyl radicals were generated by Fenton-like reaction as catalyzed by ESIONs, which was achieved under the mildly acidic tumor microenvironment, enabling the stimuli of the apoptosis and necrosis of HCC cells. This strategy demonstrated the high active-targeting capability of GE@EVs into HCC, achieving highly efficient tumor suppression both *in vitro* and *in vivo*. In addition, the as-synthesized nanoreactor could act as a desirable nanoscale contrast agent for magnetic resonance imaging, which exhibited desirable imaging capability during the sequential nanocatalytic treatment.

**Conclusion:** This application of surface-engineering EVs not only proves the high-performance catalytic therapeutic modality of GE@EVs for HCC, but also broadens the versatile bio-applications of EVs.

## Introduction

As the most frequent primary liver cancer, hepatocellular carcinoma (HCC) is the third leading cause of cancer-related deaths worldwide 1, 2. Conventional clinical treatments such as chemotherapy and radiotherapy cannot achieve satisfactory survival benefits for the most advanced HCC patients 3-5. Meanwhile, serious side effects of these traditional therapies remain to be addressed due to their off-target potential 6-8. To solve these issues, the alternative therapeutic modalities for specific and efficient treatment against HCC are urgently needed.

Extracellular vesicles (EVs) are lipid bilayer membranes that contain proteins and nucleic acids secreted by most mammalian cells including tumor cells 9-11. Various cancer cells, such as HCC cells, secrete large amounts of EVs, which are important for tumor progression and metastasis 12-16. More importantly, the typical membrane-targeting capability of various EVs could make them as favorable carriers for targeted therapy against tumors 17-20. Recently, a variety of EVs have been employed as desirable candidates in delivering siRNA or specific proteins for targeted tumor therapy 21. However, its use is mainly limited to the transport of biological macromolecules, such as proteins and RNA, to specific tumor area by intravenous injection 22-24. Furthermore, there have been very few researches on EVs acting as active-targeting therapeutic nanoreactors for combating HCC progression so far.

As an alternative to chemotherapy, the emerging nanocatalytic medicine has been developed based on the tumor microenvironment-responsive chemical reactions 25-27. It has been demonstrated that the dual enzyme-like activity of nanocatalysts could be utilized for tumor microenvironment-dependent nanotherapy, on account of toxic hydroxyl radicals (·OH) as generated by glucose oxidase (GOD) and extremely small-sized iron oxide nanoparticles (ESIONs) 28, 29. However, the main challenge of this therapeutic modality is how to avoid nonspecific delivery, which may cause decreased therapeutic efficacy and unexpected side effects. The capability of existing nanocarriers to target tumors is not satisfactory as well. Such a condition urges us to design more effective strategies to enhance the endocytosis of targeted nanoparticles, thus achieving targeted nanocatalytic therapy against tumor. To some extent, the enhanced targeted nanocarriers could be used for achieving accurate therapeutic effect, and also for reducing resistance to therapies and fatal consequences 30, 31. Additionally, nanocatalytic medicine requires relatively biodegradable and biocompatible nanocarriers for future possible clinical translation.

Herein, we report on the surface engineering of EVs as nanocarriers for the construction of targeted, sequential and biocompatible nanocatalysts, which is abbreviated as GOD-ESIONs@EVs (GE@EVs). We chose the HCC EVs in order to obtain greater membrane compatibility according to their homology. The adopted surface-loading strategy is to anchor ESIONs to EVs through receptor-ligand reaction and covalent connection between three-amino acid peptide arginine-glycine-aspartic acid (RGD) and its receptor integrins, which was abundantly present on the surface of HCC cells 32, 33. Subtly, RGD could be used as the connectome of ESIONs and EVs. Especially, it could be treated as an active target to the specific HCC tumor area. GOD loaded on EVs serves as the starting enzyme to catalyze the intracellular glucose into massive H_2_O_2_. The downstream ESIONs, which could not only be used for contrast-enhanced magnetic resonance imaging (MRI), but also transform the overproduced H_2_O_2_ within tumor region *via* Fenton-like reaction to toxic hydroxyl (·OH) radicals, thus inducing mitochondria damage and promoting tumor-cell death. More efficient treatment of sequential nanocatalysts in the HCC tumor region in a shorter period of time was achieved by enhancing the intracellular endocytosis of RGD-targeting effect and membrane fusion. Additionally, the glucose-starving reaction mediated by the nanocarriers could strengthen the inhibition rate against HCC progression and achieve synergistic therapy combined with catalyzed tumor suppression.

## Results and Discussion

### Synthesis and characterizations of GE@EVs

**Scheme 1:**
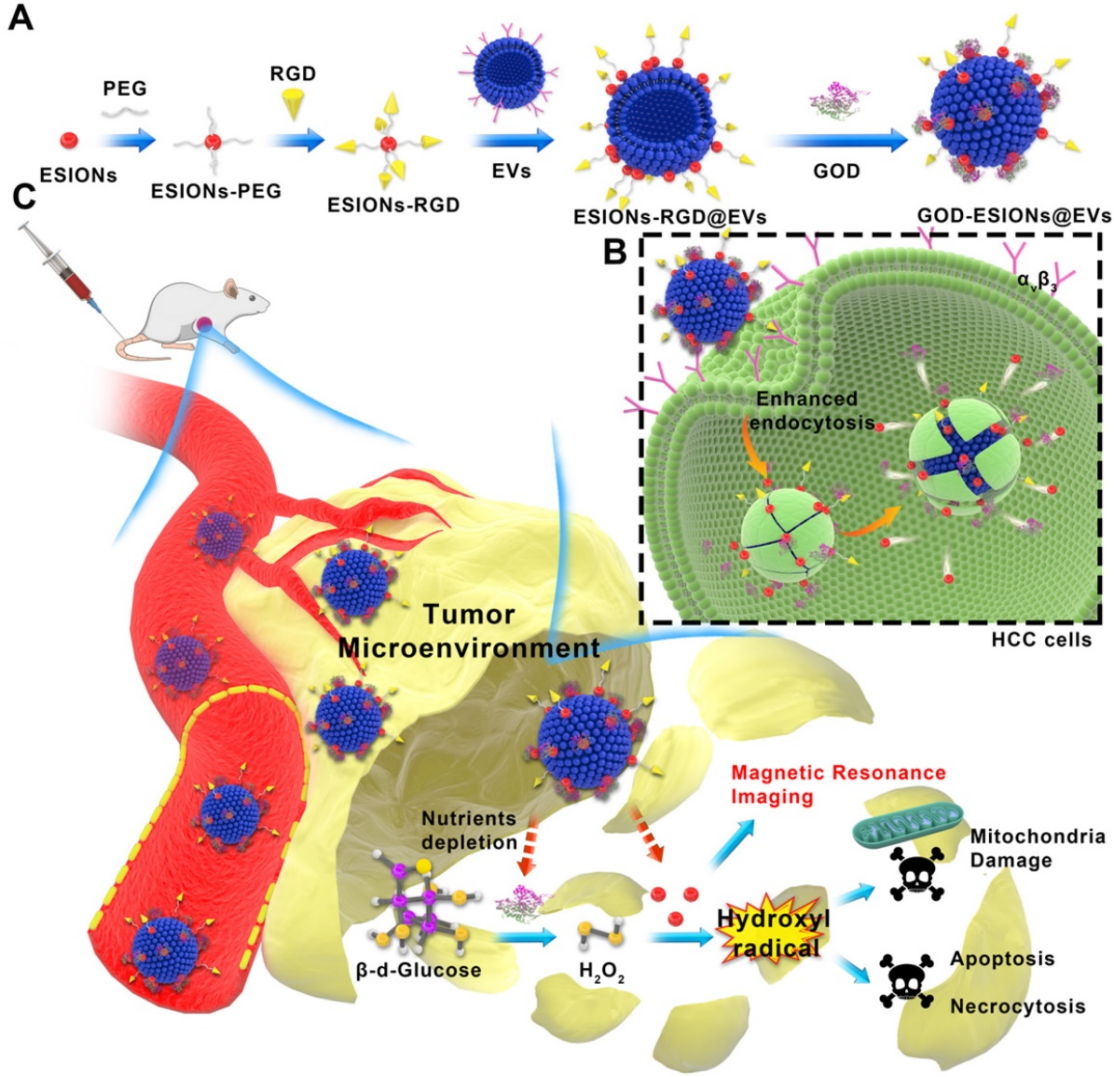
** Schematic illustration of the synthetic process of GE@EVs and sequential catalytic therapy against HCC with enhanced intracellular endocytosis.** (A) Schematic diagram for the fabrication of GE@EVs, including decoration of ESIONs and covalent linkage with GOD. (B) The scheme of the procedure of intracellular endocytosis and disintegration of GOD-ESIONs@Exo, including receptor ligand binding of RGD. (C) Schematic illustration of theranostic functions of GE@EVs, including free transportation within the blood vessel after intravenous injection, sequential catalytic therapy based on Fenton-like reaction, magnetic resonance imaging, cytotoxic effects on mitochondria and cell apoptosis and necrosis.

**Figure 1:**
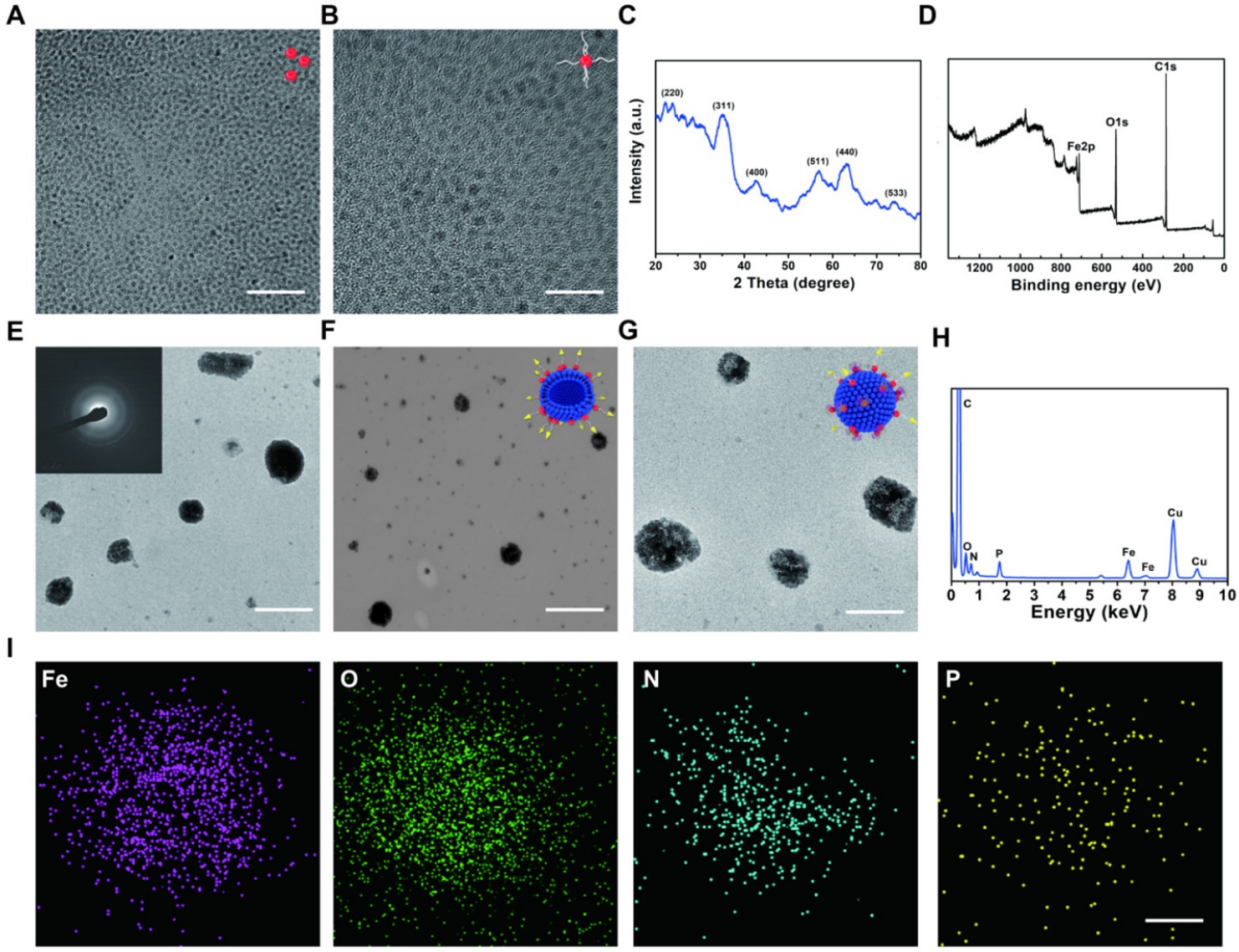
** Structural and compositional characterizations of ESIONs, ESIONs-PEG, ESIONs-RGD@EVs, and GE@EVs.** (A) TEM images of ESIONs at different magnifications before PEG decoration. (Scale bar: 20 nm). (B) TEM images of ESIONs surface-decorated by DSPE-PEG-NHS. (Scale bar: 10 nm). (C) XRD pattern and (d) XPS spectrum of ESIONs. (E-F) TEM and STEM images of ESIONs-RGD@EVs with electron diffraction (Scale bar: 100, 200 nm). (G) TEM images of GE@EVs (Scale bar: 200 nm). (H) EDS elemental analysis of GE@EVs. (I) Elemental-mapping images of GE@EVs (Fe, O, N and P elements). (Scale bar: 20 nm).

EVs as carriers were prepared from HCC Huh 7 cell lines by using ultra-centrifugation methods and then dispersed in phosphate buffer saline (PBS) 34, 35. In this way we isolated EVs, which are generally used in the experiment. This isolation method can also remove impurities as much as possible to avoid the side effects on the cells. ESIONs were fabricated *via* a facile thermal decomposition method and modified by DSPE-PEG-NHS 36, 37. RGD was covalently bounded to the PEG-modified ESIONs, which established the connection of ESIONs and EVs (**Scheme 1A**). The RGD peptide has been identified to target α_v_β_3_ integrin receptor on several tumor angiogenesis 38, 39. The application of RGD peptide in hepatocellular carcinoma can be referred to previous research 33. Enhanced endocytosis of HCC for GE@EVs is due to the presence of α_v_β_3_ receptors and membrane fusion effects on cell membranes (**Scheme 1B**). GOD was finally adsorbed to ESIONs or EVs 40. Transmission electron microscopic (TEM) images of ESIONs before and after the PEGylation showed the small diameter of 2 nm with favorable dispersibility (**Figure 1A-B**). X-ray diffraction (XRD) and X-ray photoelectron spectroscopy (XPS) patterns exhibited the specific crystal structure of ESIONs, revealing its successful synthesis (**Figure 1C-D and Supplementary Figure S1**). TEM and scanning transmission electron microscopic (STEM) of purified EVs and ESIONs-RGD@EVs were also recorded, which demonstrated the close distribution of ESIONs on EVs surface (**Figure S2a and Figure 1E-F**). EVs appear as round vesicles with a homogeneous diameter, which present a typical cup-shaped morphology composed of phospholipid bilayer. EVs were then characterized following MISEV 2018 guidelines (**Figure S2B-C**). TEM and STEM images of GE@EVs demonstrated that the spherical membrane structure was kept after the loading with ESIONs and GOD (**Figure S3 and Figure 1G**). Due to a large amount of ESIONs adhered to the surface of EVs, GE@EVs presented the appearance of spherical membrane structure with granular surface in the TEM/SEM images. Such a surface modification does not change the biofilm properties of the EVs, while preserving the biomolecules on its surface. The result of Western blot shows that the surface modification in GE@EVs does not damage the proteins expressed on the EVs. (**Figure S2C**), which confirmed the homology of GE@EVs and the pure EVs, suggesting that the adopted method herein would not damage the EVs structure.

**Figure 2:**
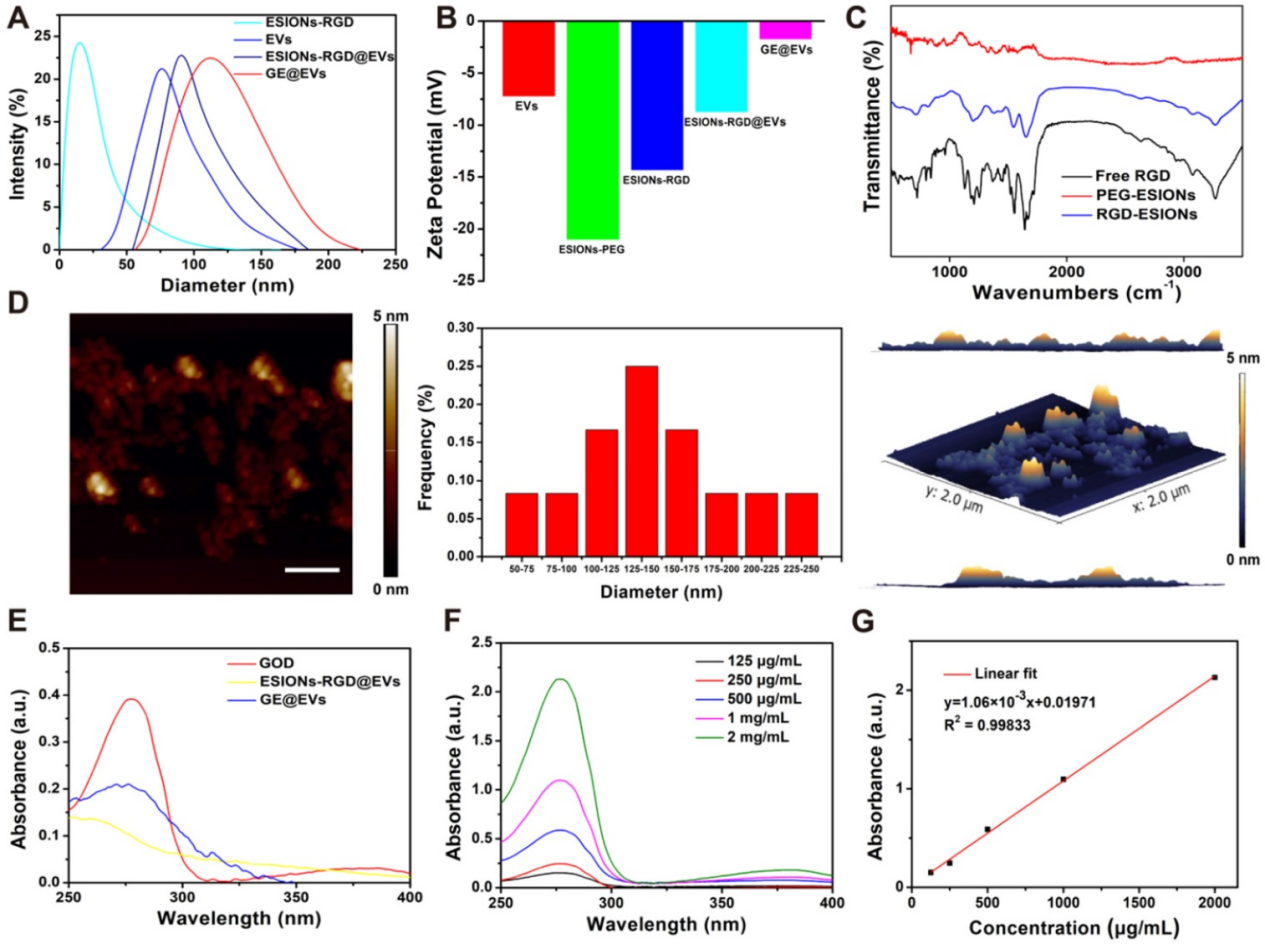
** Composition and structure characterization of GE@EVs**. (A) DLS measurements and (B) zeta potential of nanomedicines as synthesized at each step. (C) FTIR spectra of ESIONs-PEG, ESIONs-RGD and free RGD. (D) Typical AFM image of GE@EVs, including lateral size distribution analysis and AFM 3D view of area in panel. (Scale bar: 200 nm). (E) UV-vis spectra of GOD, ESIONs-RGD@EVs and GE@EVs. (F) UV-vis spectra and (G) normalized absorbance intensity at 280 nm of varied concentrations of GOD dispersed in aqueous solution (125, 250, 500, 1000 and 2000 µg/mL).

Energy dispersive spectroscopy (EDS) analysis and elemental mapping were performed to analyze the element composition of GE@EVs, which confirmed the successful loading of ESIONs on EVs (**Figure 1H-I**). In particular, the results of DLS measurements of EVs, ESIONs-RGD@EVs and GE@EVs showed their slightly increased hydrodynamic diameters from around 80 to 120 nm, which was attributed to the loading of ESIONs and GOD (**Figure 2A**). This change in particle size is due to the fact that the hydration particle size is slightly larger than the actual particle size. The zeta potential of EVs, ESIONs-RGD@EVs and GE@EVs exhibited a decrease after the loading of PEGylated ESIONs onto EVs from around -20 to -2 mV (**Figure 2B**). The Fourier transform infrared spectroscopy (FTIR) spectra of ESIONs-RGD and free RGD showed distinctive bands at 1665 and 1529 cm^-1^ (amide II, N(H)-C(O)), confirming the effective linkage of RGD 32 (**Figure 2C**).

GE@EVs could be stable for 7 days at 4 °C, which was verified according to its neglectable change in diameter both in pH 7.0 and 6.0 (**Figure S4**). Then it was easy to simulate GOD's release *in vitro* as the EVs ruptured (**Figure S5**). Atomic force microscope (AFM) measurement proved that the lateral size was statistically 142.7 nm on average, which matched well with the TEM characterization (**Figure S2D**). The increased thickness of GE@EVs on the flat surface in 3D image of AFM was probably caused by the presence of iron oxide nanoparticles. The presence of RGD was evidenced *via* the organic matter change at 1555 cm^-1^ in Raman characteristic peak 41 (**Figure S6A-B**). The efficient loading of GOD was confirmed by the ultraviolet absorption peak at 280 nm of GE@EVs when compared to ESIONs-RGD@EVs (**Figure 2E**). Characteristic ultraviolet absorption peaks of different concentration of GOD assisted to quantify the loading capability of ESIONs@EVs according to the linear fitting function (**Figure 2F-G**). By detecting the concentration of iron element and GOD in the supernatant, it was calculated that the loading amounts of GOD and ESIONs in GE@EVs were 223 and 241 µg per mg pure EVs, respectively.

### Catalytic performance of GE@EVs and *in vitro* production of hydroxyl radicals

**Figure 3:**
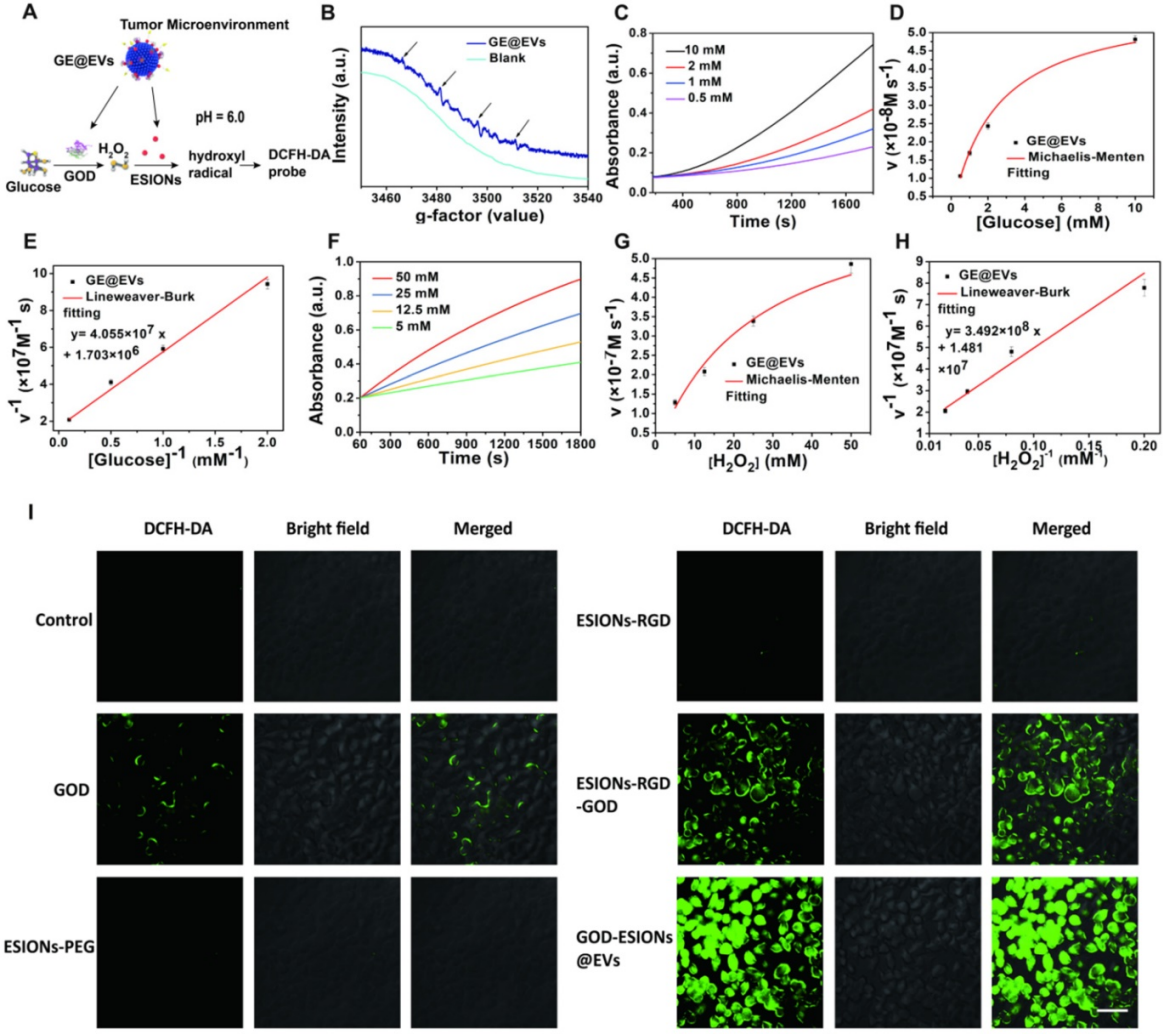
***In vitro* evaluation of nanocatalytic performances of GE@EVs and intracellular ROS production efficiency.** (A) Schematic image of nanocatalytic procedures of GE@EVs in mildly acidic condition. (B) ESR spectra of GE@EVs (10 µg/mL) with or without (blank) the addition 10 mM glucose. (C-H) Michaelis-Menten steady-state kinetics of GE@EVs. (C, F) Time-course absorbance of GE@EVs upon the addition of varied concentrations of β-D-glucose (10, 2, 1 and 0.5 mM) and H_2_O_2_ (50, 25, 12.5, and 5 mM). (D, E) Michaelis-Menten kinetics and Lineweaver-Burk plotting of GE@EVs with addition of β-D-glucose. (G, H) Michaelis-Menten kinetics and Lineweaver-Burk plotting of GE@EVs with addition of H_2_O_2_. (I) CLSM images of intracellular ROS production. (Scale bar = 50 µm).

The crucial step of nanocatalytic therapy based on Fenton reaction was the production of hydroxyl radicals (**Figure 3A**). GE@EVs firstly catalyzes β-D-glucose into H_2_O_2_ by the loaded GOD biologically. Sequentially, the co-loaded ESIONs catalyze the disproportionation of H_2_O_2_ intermediate to produce cytotoxic hydroxyl (·OH) radicals under acidic pH of tumor microenvironment, while O_2_ and H_2_O generated in a neutral environment. 5,5-dimethyl-1-pyrroline-N-oxide (DMPO), the typical nitrogen trap, was used to trap short-lived radicals in electron spin resonance (ESR) spectroscopy analysis. The presence of characteristic 1:2:2:1 hydroxyl radical signals demonstrated that the presence of glucose (10 mM) assisted the generation of considerable amount of hydroxyl radicals 42, 43 (**Figure 3B**). Comparatively, no obvious signals were observed in the ESR spectrum without the addition 10 mM glucose under identical measurement condition (blank).

Colorless 3,3',5,5'-tetramethyl-benzidine (TMB) could be oxidized to chromogenic TMB by the produced ·OH, which was detected at 650 nm using a spectrometer. Based on this mechanism, Michaelis-Menten kinetic characteristics were investigated for assessing the catalytic performances of the designed nanocatalytic system. Glucose (10, 2, 1 and 0.5 mM) and H_2_O_2_ (50, 25, 12.5 and 5 mM) were selected as the reactants, while the concentration of GE@EVs was in the assay of 100 μg/mL. The average initial velocities were calculated according to the time-course absorbance upon the addition of glucose into GE@EVs in pH = 6.0 buffer (**Figure 3C**). Based on the Beer-Lambert law, the absorbance changes would be converted into initial velocities (*V_0_*) of hydroxyl radical production 44. Then, *V_0_* was fitted with Michaelis-Menten curves against the corresponding concentration (**Figure 3D**). Furthermore, a linear double-reciprocal plot was obtained to determine the Michaelis-Menten constant (*K_M_*) and maximum velocity (*V_max_*) 29 (**Figure 3E**). The *K_M_* and *V_max_* values were calculated to be 25.19 mM and 5.87× 10^-7^ Ms^-1^ for GE@EVs nanosystem, respectively.

Meanwhile, the steady-state kinetics of GE@EVs was investigated in the assay of H_2_O_2_ supply. The time-course absorbance and Michaelis-Menten curves were plotted (**Figure 3F-G**). Linear double-reciprocal plot was obtained following Michaelis-Menten and Lineweaver-Burk equations (**Figure 3H**). The *K_M_* and *V_max_* of GE@EVs were calculated to be 2.40 mM and 5.87×10^-8^ M s^-1^, respectively. The same experiment was repeated in a neutral environment (pH = 7.4). Glucose and H_2_O_2_ supplies, as the initial substrate for the reaction, were discussed respectively. Accelerated TMB chromogenic reactions revealed the reactive oxygen species (ROS) production, but under a neutral environment, the reaction rate became much slower (**Figure S7A-F**). The amount of ROS production was limited at a low level. As a result, sufficient ROS production could be achieved in a simulated solution with tumor-microenvironment characteristics.

To verify the intracellular production of ROS in HCCs, the fluorescence probe 2′,7′-dichlorofluorescin diacetate (DCFH-DA) was used to collect and display the ·OH in confocal laser scanning microscopy (CLSM) images. There was neglectable green fluorescence in ESIONs-PEG and ESIONs-RGD-treated Huh 7 cells due to the low intracellular H_2_O_2_ level (**Figure 3I and Figure S8**). It indirectly proved the initial effect of GOD in the whole ROS production reaction. Strong green fluorescence was observed in the GE@EVs-treated cancer cells compared with pure GOD and ESIONs-RGD-GOD, which could be attributed to the enhanced endocytosis and active-targeting effect.

### *In vitro* cytotoxicity profiles and nanocatalytic anti-HCC therapy

**Figure 4:**
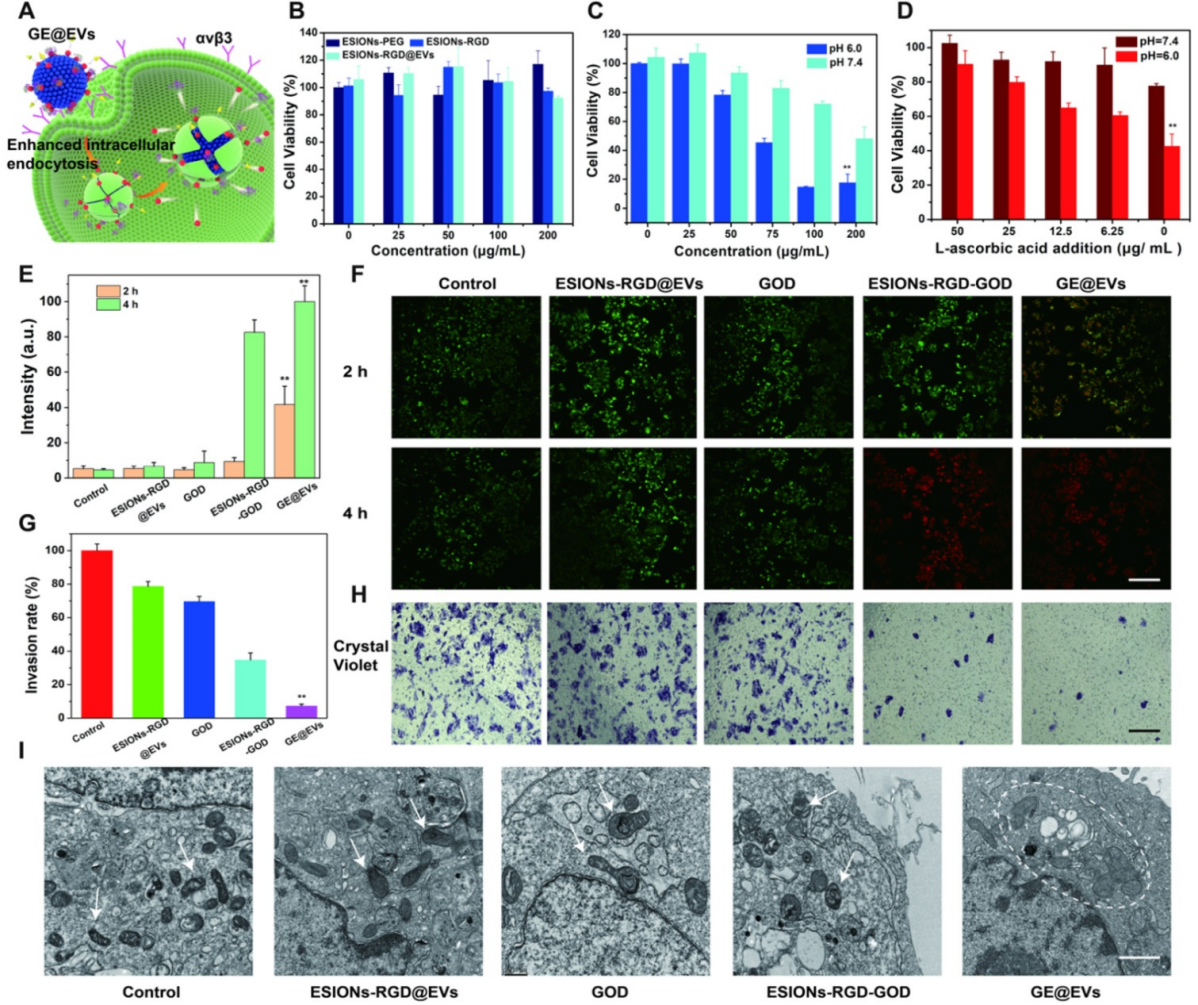
***In vitro* cytotoxicity profiles and nanocatalytic therapy against HCC Huh 7 cells. (**A) Schematic illustration of nanocatalytic therapy against Huh 7 cells. (B) Cytotoxicity of ESIONs-PEG, ESIONs-RGD and ESIONs-RGD@EVs after the incubation with Huh 7 cell lines for 24 h. (C) Relative viabilities of Huh 7 cells at different concentrations of GE@EVs in mildly acidic and neutral solution. (D) L-ascorbic acid-assisted cell rescue profiles of Huh 7 cells cytotoxicity induced by 100 µg/mL of GE@EVs. (E) Red fluorescent quantitation and (F) CLSM images of Huh 7 cells stained by calcein AM (green) and PI (red) incubated for 2 and 4 h after different treatments (Scale bar = 40 µm). (G) Invasion rate results of transwell images after different incubations. (H) Transwell images and cellular invasion profiles of Huh 7 cells after different incubations (Scale bar = 100 µm). (I) Bio-TEM images of intracellular organelles after different treatments. The white arrow points out the normal cell shape, and white dotted line circle represents the swollen and deformed mitochondria and golgi bodies (Scale bar = 1 µm). *P < 0.05 and **P < 0.01.

The catalytic effect of GE@EVs could be induced due to intracellular endocytosis mediated by RGD and membrane fusion (**Figure 4A**). As schematically designed, RGD on the surface of GE@EVs could specifically bind to the receptors (integrin α_ν_β_3_) on Huh 7, and then GE@EVs was engulfed and degraded to generate downstream products. The cytotoxicity profiles of the ESIONs-RGD@EVs were evaluated *via* a typical cell-counting kit-8 (CCK-8) assay (**Figure 4B**). The results were consistent with the confocal microscopy results, which exhibited that the loaded GOD played an initial and important role in the cytotoxic reaction by comparison with GE@EVs and ESIONs-RGD@EVs groups. The cell viabilities were much lower under the acidic condition than that under neutral condition (**Figure 4C**), demonstrating the unique response to tumor acidity. The relative cell viabilities were 89.0, 77.3, 63.4, 61.2, 40.1% *vs*. 99.5, 90.6, 88.9, 84.3, 77.8% with pH values at 6.0 and 7.4 of elevated GE@EVs concentrations, respectively. In principle, the cytotoxicity towards Huh 7 HCC cells could be rescued by the addition of L-ascorbic acid, which could combine and eliminate ·OH. L-ascorbic acid at a series of concentrations was added into the solution after the Fenton-like catalytic reaction. The increase of relative cell viability (the whole GE@EVs at 100 μg/mL) against the corresponding L-ascorbic acid concentrations confirmed such a rescue effect (**Figure 4D**), further verifying the oxidative therapeutic effect of GE@EVs.

To observe the viable and dead cell proportion visually, Huh 7 cells were stained with calcein acetoxymethyl ester (calcein-AM) and propidium iodide (PI) solution after co-incubation with PBS, ESIONs-RGD@EVs, GOD, ESIONs-RGD-GOD and GE@EVs for 2 and 4 h, respectively (**Figure 4E-F**). Cell apoptosis after nanocatalytic therapy was further confirmed by CLSM imaging. Irreversible damage of tumor cells was caused by GE@EVs in 2 h at a concentration of 100 μg/mL according to the red fluorescence quantification (**Figure 4E**). Importantly, GE@EVs achieved a higher therapeutic effect than ESIONs-RGD-GOD, which demonstrated the enhanced anticancer effect *via* EVs-mediated endocytosis. These results matched well with the CCK-8 profiles, which further verified that the therapeutic effect was caused by the cytotoxic hydroxyl radicals *in-situ* produced by GE@EVs.

To further explore how ROS affected invasiveness of HCC cells, the transwell invasion assay of Huh 7 cells was performed. The experimental groups were the same as the aforementioned live/death cell-staining experiments. The cancer cells were incubated with GE@EVs in hypoxic condition to simulate the anaerobic environment of solid tumors for 12 h, then stained with crystal violet in methanol. The microscopic images exhibited that GE@EVs could effectively inhibit the invasiveness of HCC Huh 7 cells (**Figure 4H**). The corresponding invasion rate results confirmed this result (**Figure 4G**). The minimum cell count was observed in GE@EVs group. In addition, the internal conditions of cells affected by hydroxyl radical were observed by bio-TEM microscopy. The intracellular organelle of Huh 7 cells was shown in bio-TEM image after co-incubation with GE@EVs for 4 h (**Figure 4I**). Swollen and wrinkled mitochondria were observed in the GE@EVs group (white dotted circle). Other organelles, such as golgi bodies, also underwent vacuolar changes due to irreversible damage by hydroxyl radicals. Flow cytometric analysis revealed the apoptosis and necrosis of Annexin V-FITC/PI-stained Huh 7 cells after different treatments (**Figure S9**).

### Enhanced endocytosis and active targeting of GE@EVs to HCC cells

**Figure 5:**
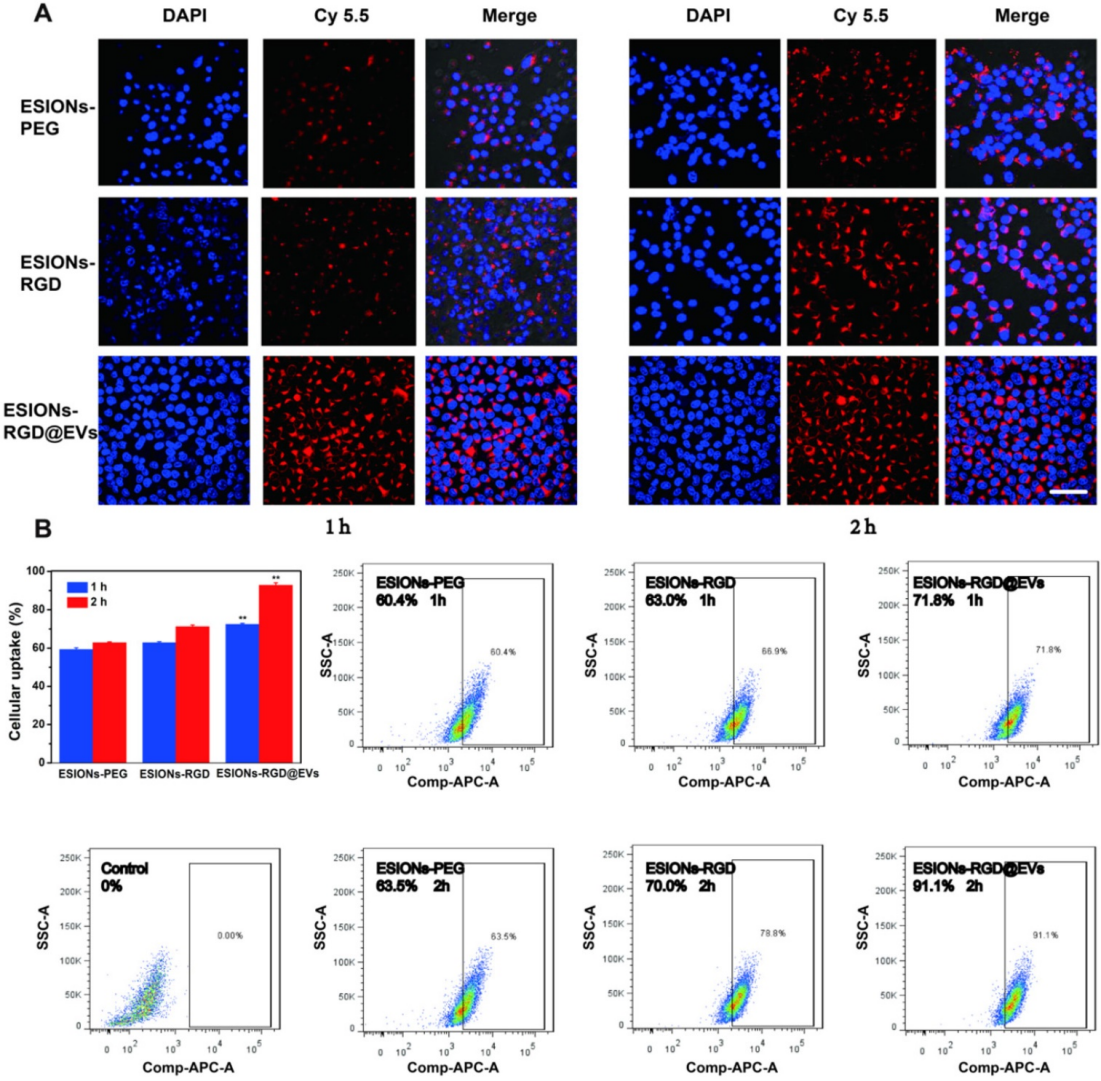
** Enhanced intracellular endocytosis of GE@EVs.** (A) CLSM analysis of Huh 7 cellular uptakes of different agents marked by Cy5.5 at 1 or 2 h (Scale bar = 50 µm). (B) Fluorescent quantitation and flow cytometry analysis of Huh 7 cells incubated with different agents for 1 or 2 h. *P < 0.05 and **P < 0.01.

Through exposed RGD on the opposite side of iron oxide, active targeting could be achieved on the surface of EVs, which made the GE@EVs obtain enhanced intracellular endocytosis (**Figure 4A**). Active-targeting effect of RGD and homogeneity of membrane structure between EVs and HCC cell were both responsible for the enhanced endocytosis of GE@EVs. In order to explore the process of endocytosis, Cy5.5 was used to connect the ESIONs particles, which could be excited at 675 nm for emitting luminescence. CLSM images exhibited that the maximum endocytosis was achieved in ESIONs-RGD@EVs group (**Figure 5A**). The fluorescence intensity of ESIONS-RGD group was obviously stronger than the ESIONs-PEG group at both 1 and 2 h, which verified the active targeting ability of RGD molecules. The fluorescence intensity of ESIONs-RGD@EVs at 1 h was much higher even than ESIONS-RGD group at 2 h, indicating the enhanced membrane fusion effect. As a naturally desirable nanocarrier, EVs could enhance the targeting capability through membrane fusion and improve endocytosis accordingly. Since GOD is considered to be the starting drug for the entire system, and is generally not considered to have the ability to target, we set groups as ESIONs-PEG, ESIONs-RGD, and ESIONs-RGD@EVs. We used the grouping design to validate the active targeting ability of RGD molecules and also to validate the enhanced endocytosis of EVs nanocarrier.

Fluorescence quantitative and flow cytometry analysis of Cy5.5 at 1 and 2 h verified this point as well (**Figure 5B**). After co-incubating with nanosystem for 1 h, the fluorescence value of Huh 7 endocytosis increased from 60.4% in ESIONs-PEG group to 71.8% in ESIONs-RGD@EVs group. The results after 2 h co-incubation were more significant (63.5%-91.1%). The enhanced intracellular endocytosis verified the increased ROS production, which could be the evidence of GE@EVs active targeting. This enrichment effect provides the bases of the subsequent evaluation on active targeting and therapeutic effect of GE@EVs against HCC *in vitro* and *in vivo*.

### *In vivo* MR imaging and nanocatalytic therapeutics of GE@EVs for HCC on HCC tumor xenograft

**Figure 6:**
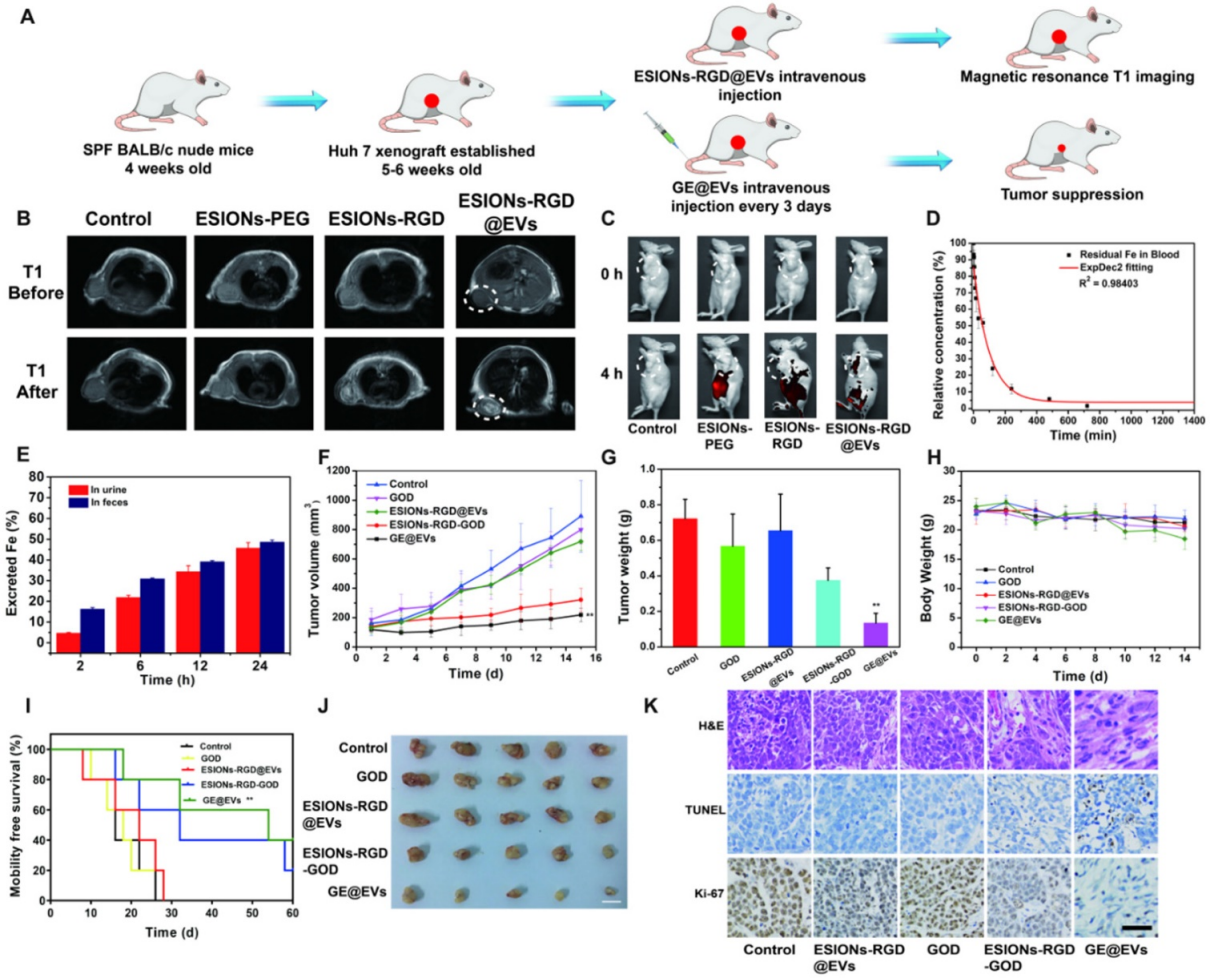
***In vivo* pharmacokinetic analysis, diagnostic imaging and therapeutic efficacy for HCC tumor-bearing mice.** (A) Schematic illustration of Huh 7 tumor xenograft establishment, GE@EVs therapeutic procedure, and MR imaging. (B) MR images of tumor-bearing mice in control, ESIONs-PEG, ESIONs-RGD and ESIONs-RGD@EVs groups under *T_1_* (0, 2 h) mode. (C) *In vivo* Fluorescence imaging of mice treated with PBS, ESIONs-PEG, ESIONs-RGD and ESIONs-RGD@EVs marked by Cy 5.5. (D) Blood-circulation lifetime of GE@EVs after intravenous injection into mice (n = 3). (E) Accumulated Fe (in feces and urine) excretion out of the mice body after the injection of GE@EVs for different durations (2, 6, 12, 24, 36 and 48 h). (F) Time-dependent tumor-growth curves of nude mice (n = 5, mean ± s.d.) after different treatments, including control, free GOD, ESIONs-RGD@EVs, ESIONs-RGD-GOD, and GE@EVs. (G) Tumor weights of mice at 28 days after the treatments of each group. (H) Time-dependent body-weight curves after different treatments. (I) Survival curves of mice after various treatments as indicated in each group. (J) Digital photographs of the dissected tumors of each group (Scale bar: 1 cm). (K) H&E staining, TUNEL staining, and antigen Ki-67 immunofluorescence staining for pathological changes in tumor tissues from each group and cellular proliferation (all scale bars: 100 µm). *P < 0.05, **P < 0.01.

Previous studies of ESIONs have proved that the ultra-small iron oxide nanoparticles were suitable for T_1_-weighted MRI of tumors 45, 46. However, the related applications are limited, because diagnosis and treatment are not integrated into the same nanosystem. Also, previous catalytic treatment systems using Fenton-like reactions often require inorganic substances as nanocarriers, which are less biocompatible than membrane carriers of EVs 47, 48. According to the nanocatalytic efficacy on inducing HCC cytotoxicity and high active-targeting ability *in vitro*, we speculated that the enrichment in HCC site and high therapeutic performance could be achieved *in vivo*. To verify the *in vivo* performance, the HCC Huh 7 tumor xenografts were built on specific pathogen-free BALB/c nude mice, which were used to analyze the tumor enrichment and tumor suppression, respectively (**Figure 6A**). ESIONs-RGD@EVs was initially evaluated to be biocompatible on healthy Kunming mice (**Figure S10-11**). MR measurements of GE@EVs *in vitro* were then analyzed for further MR imaging in mice (**Figure S12**). Due to the specific feature of ultrasmall particle size of the loaded ESIONs, more specific guidance and monitoring could be achieved than the modified ESIONs. Residual ESIONs in the tumor tissue could be used as the contrast agents for real-time MR imaging to monitor HCC progression during the treatment due to its paramagnetic characteristics (**Figure 6B**). The white circles in *T_1_* mode showed a more pronounced diagnostic MR image for the HCC imaging as compared to the other groups. ESIONs in the blood flowed to the tumor site, which were then specifically ingested by HCC cells. Because ESIONs are featured with paramagnetic properties, it can enhance the T1 signal in tumor tissue, which could be used as a potential contrast agent for therapeutic guidance and monitoring.

Furthermore, fluorescence imaging of Huh 7 tumor-bearing mice *in vivo* was presented to figure out whether satisfactory enrichment effect could be achieved in tumor region (**Figure 6C**). Distinctly, EVs could assist to transport the designed nanocatalysts to the sub-axillary tumor area according to the fluorescence quantitative analysis, reflecting the active-targeting ability of the designed nanocatalysts (**Figure S13**). Such enrichment was observed more clearly when organs were removed from the mice, and the distribution of organs was observed as well (**Figure S14A-B**). The half-time of GE@EVs in blood circulation was calculated to be 0.93 h (**Figure 6D**). Systematic excretion assay demonstrated the easy excretion of GE@EVs out of the mice body* via* the urine and the feces (**Figure 6E**). The tumor-burdened mice were divided into five groups (n = 5 each group) for the following therapeutic evaluation, including control, ESIONs@EVs, GOD,) ESIONs-RGD-GOD, and GE@EVs (10 mg kg^-1^ dose). GE@EVs could suppress the tumor progression within 200 mm^3^ with the inhibition rate of 75.5%. Comparatively, the tumors in groups that could not produce hydroxyl radical grew quickly (**Figure 6F**). Free GOD or ESIONs-RGD@EVs could not suppress the HCC growth in principle. Meanwhile, GE@EVs benefited the therapeutic mice to get the minimum tumor weight of 0.135 g (**Figure 6G**). During the treatments, all the mice weight showed little fluctuations (**Figure 6H**), demonstrating that the impact of intravenous injection of the agents on the general health of mice was negligible. To further explore the effect of GE@EVs on the long-term survival time of tumor-bearing mice, we started again and extended the observation period. Survival analysis diagram intuitively reflected the survival benefits of GE@EVs in 60 days (**Figure 6H**). Comparable therapeutic effect of GE@EVs was observed in *ex vivo* tumors after the treatment (**Figure 6J**). It was worth noting that apparent HCC-inhibiting effect was also been achieved in the ESIONs-RGD-GOD group. However, GE@EVs exhibited a higher tumor-suppressing efficiency as contributed by the satisfactory targeting-capability of EVs as nanocarriers of sequential nanocatalysts.

Tumor sections were stained with hematoxylin and eosin (H&E), TdT-mediated dUTP nick-end labeling (TUNEL) and Ki-67 antibody to observe pathological characteristics and potential HCC-suppression mechanisms of tumor tissues (**Figure 6K**). Significant apoptosis and necrosis of Huh 7 cells were viewed because of the damage caused by hydroxyl radical. Compared to other groups, H&E and TUNEL staining images of ESIONs-RGD-GOD and GE@EVs groups exhibited obvious therapeutic effect. Ki-67 antibody staining was applied to assess the *in vivo* proliferative activities. Stronger suppression on cell proliferation was observed in ESIONs-RGD-GOD and GE@EVs groups, which suggests that *in-situ* enhanced catalytic reactions could inhibit HCC growth and progression.

## Conclusions

In summary, a high-performance EVs-based theranostic nanoreactor was constructed for HCC-specific treatment. As a paradigm, simultaneous surface-loading strategy of extremely small-sized iron oxide nanoparticles and GOD enzyme on EVs exhibited desirable suppressive effect on tumor growth. Highly toxic hydroxyl radicals produced by sequential Fenton nanocatalysis resulted in significant death of HCC cells. In addition, the tumor microenvironment-specific therapeutic strategy was combined neatly with the active targeting EVs system. New therapeutic strategies for HCC were usually confined to monoclonal antibody and the combination of chemotherapy drugs 49, 50. However, the therapeutic efficacy was often uncertain, and the side effects of chemotherapy drugs are often not negligible 51-55. The enhanced intracellular endocytosis made the catalytic therapeutic effect of hydroxyl radicals in-situ relatively stronger applicability. As a natural extracellular vesicle secreted by living cells, EVs have extraordinary advantages in biocompatibility and biodegradability 56-58. Compared with previous studies on EVs mainly about the contained RNA, we focused more on its homology with tumor cell membrane, and increased its tumor regional enrichment by using active targeting molecule RGD 59, 60. Our strategy has broadened the biomedical applications of EVs-based transportation nanoplatforms for combating HCC by elaborate surface engineering on EVs. To the best of our knowledge, this is the first report on surface modification of HCC-derived EVs, which were endowed with efficient targeting capability and catalytic therapeutic efficacy against HCC. Additionally, the unique paramagnetic property of iron oxide nanoparticles also gained high potential for real-time monitoring of the treatment process.

## Methods

### Materials

Ingredients for the synthesis of iron oxide such as 90% oleic acid, 98% iron chloride hexahydrate (FeCl_3_·6H_2_O), 95% sodium oleate, oleyl alcohol, 90% oleylamine, ethanol and chloroform (CHCl_3_) were purchased from Sigma-Aldrich. DSPE-PEG-NHS, RGD (Arg-Gly-Asp) peptides and fluorescent dyes containing DCFH-DA, AM/PI and Cy5.5 were purchased from Aladdin. HCC Huh 7 cells were bought from the Cell Bank of the Committee of Type Culture Collection of Chinese Academy of Sciences.

### Synthesis of GE@EVs

Iron-oleate complex was firstly synthesized according to the previously report 61. For the synthesis of ultra-small iron oxide nanoparticles, iron-oleate complex (1.8 g, 2 mmol), oleic acid (0.57 g, 2 mmol), and oleyl alcohol (1.61 g, 6 mmol) were dissolved in diphenyl (10 g) ether at room temperature. The mixture was heated to 250 °C at a constant heating rate of 10 °C/min and then maintained at this temperature for 30 min under argon atmosphere. After the reaction, the mixture containing the nanoparticles was rapidly cooled to room temperature, and acetone (50 mL) was added to precipitate the ESIONs. The produced ESIONs were resuspended into trichloromethane (CHCl_3_). ESIONs CHCl_3_ dispersion (2 mL, 10 mg/mL) was diluted to 20 mL with the same solvent. DSPE-PEG-NHS (35 mg) was then added into system for ultrasonic dispersion with argon used as a shielding gas. Next, the mixture was heated slowly to 70 °C and kept at this temperature for 5 h. After the reaction, ethanol was added to precipitate PEG-coated ESIONs, which were collected by centrifugation and redispersed in distilled water, and the remaining ethanol was removed by evaporation. After further washing and re-dispersion in PBS, cyclic RGD (Arg-Gly-Asp) peptides (50 mg) were added to covalently attach to the surface 32, and the solution was stirred for another 24 h at room temperature.

EVs were isolated by ultra-centrifugation according to the previously described standard methods 32. In brief, Huh 7 cells were grown until they reached a confluency of 80-90% in the FBS-depleted RPMI media. Next, the media was collected and centrifuged at 800 g for 5 min, and then by a centrifugation of 2,000 g for 10 min to remove cellular debris. The media was then filtered using a 0.2-mm pore filter, and then ultracentrifuged at 100,000 g for 2 h at 4 °C. The obtained EVs were washed with 30 mL PBS, followed by ultracentrifugation at 100,000 g for 2 h at 4 °C. Afterwards, the EVs were resuspended in PBS. EVs used for protein extraction were resuspended in 250 μl of lysis buffer. EVs proteins weight was obtained by quantitative results using BCA detection kit.

EVs (2 mg/mL) were added into the solution of ESIONs-RGD and stirred for 24 h. ESIONs-RGD@EVs solution was centrifuged and rinsed with PBS to remove dissociative ESIONs-RGDs. GOD (10 mg) was added to ESIONs-RGD@EVs solution under mild magnetic stirring for surface adsorption for 24 h 40. The change of absorbance of residual liquid before and after centrifugation was used to calculate the mass of GOD adsorbed. The centrifugal velocity used here was 13000 g, and the change in absorbance was easy to detect due to the relatively excessive GOD added. The GE@EVs could be acquired and purified by centrifugation. Stirring and centrifugation experiment were carried out in a cool and relatively dark environment at approximately 20 °C. The synthetic GE@EVs was stored at 4 °C for a short time during the whole experiment.

### Characterization

TEM and EDS were analyzed for microstructure and composition on the JEM-2100F electron microscope operated at 200 kV. STEM and element mapping scanning were obtained on field-emission Magellan 400 microscope under the FEI Company. XRD pattern was recorded on a Rigaku D/MAX-2200 PC XRD system. XPS spectrum was recorded on ESCAlab250 (Thermal Scientific). DLS and Zeta potential were tested on Zetasizer Nanoseries (Nano ZS90, Malvern Instrument Ltd.). AFM images were collected on the Veeco DI Nanoscope Multi Mode V system. UV-vis-NIR absorption spectra were recorded on UV-3101 Shimadzu UV-vis-NIR spectrometer. FTIR pattern was recorded for the analysis of chemical bonds. The quantitative analysis of Fe element was conducted on inductively coupled plasma-optical emission spectrometry (ICP-OES, Agilent 725, Agilent Technologies). Raman spectroscopy pattern was collected on a DXR Raman microscope (Thermal Scientific, USA). ESR spectrum was measured using DMPO as the nitrogen trapping agent by Bruker EMX1598 spectrometer.

### Stability test of GE@EVs

GE@EVs was centrifuged and resuspended in 4 °C in PBS in order to prove the preservability at low temperature. Every 24 h, DLS was used to record the particle size of GE@EVs. The stability was compared with the pure EVs. The particle size was measured within 7 days after oscillation, at least 3 times for each round, and the results were averaged. The results of DLS were used to assess the stability of GE@EVs.

### Characterization of EVs

The prepared EVs were fixed with 2% glutaraldehyde (Sigma, USA). After 30 min, 10 µL of fixed samples was pipetted onto copper grids with carbon-coated formvar film and incubated for 10 min. Grids were washed three times with ddH_2_O and the excess liquid was removed by blotting. Micrographs of sEV were obtained with a Transmission electron microscopy (JEM-2100F electron microscope). For particle size analysis, dynamic optical diffraction was used to fit the curve.

Western blots were performed in the standard fashion using Mini-PROTEAN 4-20% SDS-PAGE gels and the Trans-Blot Turbo Transfer System (Bio-Rad). The following antibodies were used: anti-CD9 mouse antibody (Abcam, [MEM-61] (ab2215)), anti-CD81 mouse antibody (Abcam, [M38] (ab79559)), anti-calnexin rabbit antibody (Abcam, (ab22595)), anti-RGD rabbit antibody (Abcam, (ab224465)).

### Michaelis-Menten kinetics

Catalytic velocity of GE@EVs (100 μg/mL) was monitored by 3.2 mM TMB according to the chromogenic reaction (λ = 650 nm) of a series H_2_O_2_ or β-D-glucose concentration. NaAc buffer solution (20 mM pH= 5.2) was used to control the pH environment and fill the final volume in the 96-well plate. The Michaelis-Menten kinetic curve of GE@EVs was calculated by plotting the respective initial velocities against H_2_O_2_ and β-D-glucose concentrations under a slightly acidic and neutral environment. The respective Michaelis-Menten constant (*K_M_*) and maximal velocity V_max_ were fitting and calculated *via* the Lineweaver-Burk plotting.

### Intracellular ROS production

1 × 10^5^ of HCC Huh 7 cells were digested and resuspended into 1 mL high-glucose DMEM (10% FBS containing) medium and subcultured into CLSM-exclusive culture disk for further 12 h incubation. Subsequently, the medium was replaced by 1 mL of high-glucose DMEM (at pH 7.4 or 6.0) containing 10 μg mL^-1^ of GOD, ESIONs-PEG, ESIONs-RGD, ESIONs-RGD-GOD and GE@EVs after twice rinsing. DCFH-DA was used to capture and mark the intracellular hydroxyl radical. DCF (λ_ex_ = 480 nm, λ_em_ = 525 nm) was observed on CLSM (FV1000, Olympus Company, Japan). Fluorescence quantification was recorded respectively.

### *In vitro* CCK-8 assay

Huh 7 cells (3000 cells per well) were inoculated into a 96-well plate and incubated for 12 h to wait for the attachment of cells. The medium of the 96-well plate was discarded, and the plate was rinsed with PBS twice. Then, ESIONs-RGD@EVs and GE@EVs at elevated concentrations of 25, 50, 75, 100 and 200 μg/mL were added into the 96-well plate with high-glucose DMEM containing 10% FBS. The mediums were discarded, and the plate was rinsed with PBS carefully after 24 h incubation at 37 °C. 100 μl 10% Cell Counting Kit-8 containing high-glucose DMEM medium was added into each well. CCK-8 was used to evaluate the cell viability in 4 h by a characteristic peak at 450 nm in the spectrometer.

### L-ascorbic acid rescue assay

The Huh 7 cells were inoculated into a 96-well plate in the same manner as mentioned before. After 4 h incubation with GE@EVs, the cell mediums were discarded and replaced with L-ascorbic acid and 10% FBS containing high-glucose DMEM medium. The concentrations were set to be 50, 25, 12.5 and 6.25 μg/mL. CCK-8 was used to evaluate the cell viability in 4 h.

### Detection of living and dead cells

1 × 10^5^ of HCC Huh 7 cells were digested and resuspended into 1 mL high-glucose DMEM containing 10% FBS medium and subcultured into CLSM-exclusive culture disk for further 12 h incubation. After treatment and co-incubation for 1 or 2 h, calcein-AM/PI staining reagents were used to stain dead cells as red fluorescence (λ_ex_ = 535 nm, λ_em_ = 617 nm) and the viable cells as green fluorescence (λ_ex_ =490 nm, λ_em_ = 515 nm). After 15 min of incubation, the staining solution was removed and rinsed by PBS twice and the samples were subsequently visualized by CLSM. Flow cytometric analysis for evaluating the apoptosis and necrosis of Annexin V-FITC/PI-stained Huh 7 cells were also performed for analysis after different treatments for 12 h.

### Transwell invasion assay

Suitable Matrigel was filled into the upper chamber of the transwell apparatus with 8 μm pore size membrane. After the Matrigel solidified, 1 × 10^5^ Huh 7 cells were seeded onto the Matrigel and incubated with grouping solutions at 37 °C overnight. Membranes coated with Matrigel were swabbed with a cotton swab and fixed with 100% methanol for 10 min. We ensured the same number of cells when preparing the cell suspension and adding the transwell chamber. And we did the experiment under the same experimental conditions. The membrane with cells was soaked in crystal violet for 3 h and then washed with deionized water. The number of cells attached to the lower surface of the polycarbonate filter was observed and counted under the light microscope.

### Mitochondria observation

GE@EVs (100 μg/mL) was incubated with the Huh 7 cells for 12 h at 37 °C. Cell scrapers were used to separate the cells from its base. 2.5% glutaraldehyde fixing solution was applied to fix the cellular structure. Bio-TEM was applied to observe and evaluate the damage and destruction of Huh 7 cells.

### Intracellular endocytosis by CLSM observation

ESIONs-PEG, ESIONs-RGD, and ESIONs-RGD@EVs (100 μg/mL) were stirred with fluorescein isothiocyanate (FITC, 2mg, Sigma-Aldrich, Shanghai, China) at 37 °C overnight. 1×10^5^ of HCC Huh 7 cells were digested and resuspended into 1 mL high-glucose DMEM (10% FBS containing) medium and subcultured into CLSM-exclusive culture disk for further 12 h incubation. The culture media was then discarded and replaced with FITC-ESIONs-PEG, ESIONs-RGD, and ESIONs-RGD@EVs in high-glucose DMEM (10% FBS containing). DAPI (Beyotime Biotechnology) was added into the dish to stain the cell nucleus for 15 min before CLSM observation. After the dye was washed clearly, the Huh 7 cells are immobilized with paraformaldehyde. The dyed samples could be subsequently visualized by CLSM.

### *In vivo* toxicity assay

The animal experiments were carried out in compliance with the Regional Ethics Committee for Animal Experiments, and the care and use regulations were approved by ethics committee of Second military medical university. SPF experiment female Kunming mice (~ 20 g, 4 weeks old) were purchased from Shanghai SLAC Laboratory Animal Co. Ltd and taken good care of. The mice were randomly divided into 8 groups (n = 3) and were injected with ESIONs-RGD@EVs dissolved in PBS at the concentration of 0, 5, 10 and 20 mg/kg, and were fed for 7 and 28 days to evaluate the acute and chronic toxicity. The body weight was measured recorded every 2 days before sacrificed with CO_2_ asphyxiation at given days. The blood samples of mice were collected for complete blood panel test and serum biochemistry assay. The major organs (heart, liver, intestine, spleen, lung and kidney) of the mice were further dissected, which were then fixed in 10% formalin and stained with the hematoxylin and eosin (H&E) for further histological analysis.

### *In vivo* blood circulation

To figure out the blood circulation condition of ESIONs-RGD@EVs, SPF female Kunming mice were intravenously injected with ESIONs-RGD@EVs dissolved in PBS (n = 3). At predetermined time intervals, blood sample (10 μl) was collected. Heparin sodium (50 unit/mL) was used to prevent blood clotting. The concentration of Fe was then measured by ICP-AES. The *in vivo* half-life in blood of ESIONs-RGD@EVs was calculated by an ExpDec2 pharmacokinetic fitting model.

### *In vivo* metabolism

To investigate the downstream metabolism process of ESIONs-RGD@EVs *in vivo*, ESIONs-RGD@EVs (100 μl, [Fe] =10 mg/kg) was injected into SPF female Kunming mice. The urine and feces of mice were collected at varied time intervals (2, 6, 12 and 24 h). The Fe content in the collected sample was determined by ICP-AES.

### *In vivo* optical imaging

Huh 7 tumor-bearing nude mice were injected with Cy 5.5-ESIONs-PEG, ESIONs-RGD, and ESIONs-RGD@EVs (100 µL) and imaged by using the Maestro *in vivo* optical imaging system (Cambridge Research & instrumentation, Inc) after 4 h. The organs were taken from the mice for biodistribution analysis. Fluorescence quantitative data collection was completed as well. The excitation light wavelength of optical fluorescence was 650 nm. The fluorescence imaging results were analyzed with Visque Clevue software.

### *In vivo* catalytic therapy against HCC tumor growth

Female BALB/c nude mice (~ 20 g) of 4-week old were purchased from shanghai SLAC Laboratory Animal Co., Ltd. Typically, Huh 7 cells were subcutaneously injected to the alar skin of female BALB/c nude mice to establish the tumor xenograft. When the tumor volume reached around 100 mm^3^, the mice were divided into 5 groups (n = 5 for each group) including (1) control, (2) ESIONs-RGD@EVs, (3) GOD, (4) ESIONs-RGD@EVs, and (5) GE@EVs. Saline solution was injected intravenously as the negative control group. The volume of the tumor was measured by the Vernier caliper every day for 14 days after the corresponding experiments. The individual tumor volume was calculated as the following equation: Tumor volume = longest diamater × shortest diameter^2^ / 2. Survival intervals were observed for 60 days, which were performed on the other 5 groups of mice as mentioned. The tumors and important organs were dissected and weighed after the corresponding treatments. Tissue samples were fixed in 10% formalin after the therapy. H&E, TUNEL and Ki-67 were applied for histological analysis.

### Magnetic resonance imaging

For MR imaging *in vitro*, centrifuge tubes containing contrast agent solutions (1 mL in each tube) were placed in a coil in a clinic MR scanner (DiscoveryMR750, GE Medical System, LLC, USA).

The *in vivo* tumor model was established as mentioned. The nude mice were intravenous injected with Fe dose of 10 mg·kg^-1^ ESIONs-PEG, ESIONs-RGD, and ESIONs-RGD@EVs in MR physiological saline solutions. MR test was performed under *T_1_* mode to compare the images after 2 h. The experimental parameters of T_1_-weighted fast-recovery spin-echo sequence were set as follows: Slice = 8 mm, Space = 0.2 mm, Auto TR = 3000, Freq. FOV=6.0, Phase fov = 0.8, Slice Thickness=1.8.

### Statistical analysis

Data were expressed as means ± standard deviation (SD)/standard error (SE) and were compared by means of an unpaired Student's t test or Mann-Whitney U test. All the statistical analyses were conducted using the Graphpad software (version 5.0).

## Supplementary Material

Supplementary figures and tables.
