# Supplementary material for: Extracellular-vesicles delivered tumor-specific sequential nanocatalysts can be used for MRI-informed nanocatalytic Therapy of hepatocellular carcinoma
Source: Theranostics. 2021 Jan 1;11(1):64–78. doi: 10.7150/thno.46124 (PMC7681081; doi:10.7150/thno.46124)
Supplement: Supplementary file 1 — Supplementary figures and tables. [file thnov11p0064s1.pdf]

Supporting information for

Extracellular Vesicles-Delivered Tumor-Specific Sequential Nanocatalysts can be used for MRI-Informed Nanocatalytic Therapy of Hepatocellular Carcinoma

Han Wu[1*], Hao Xing[1*], Meng-Chao Wu[1], Feng Shen[1], Yu Chen[2✉], and Tian Yang[1✉]

1. Department of Hepatobiliary Surgery, Eastern Hepatobiliary Surgery Hospital, Second Military Medical University, Shanghai 200438, P. R. China

2. School of Life Sciences, Shanghai University, Shanghai 200444, P. R. China

*These authors contributed equally to this work.

✉ Correspondence authors: Tian Yang (email: yangtianehbh@smmu.edu.cn) and Yu Chen (email: chenyuedu@shu.edu.cn).

Supplementary figures:

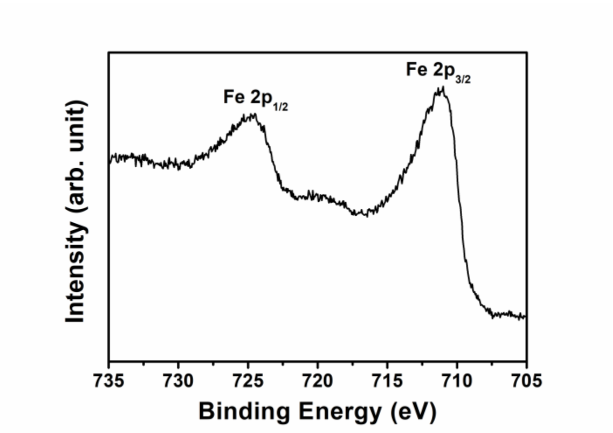

Figure S1. XPS spectrum of ESIONs.

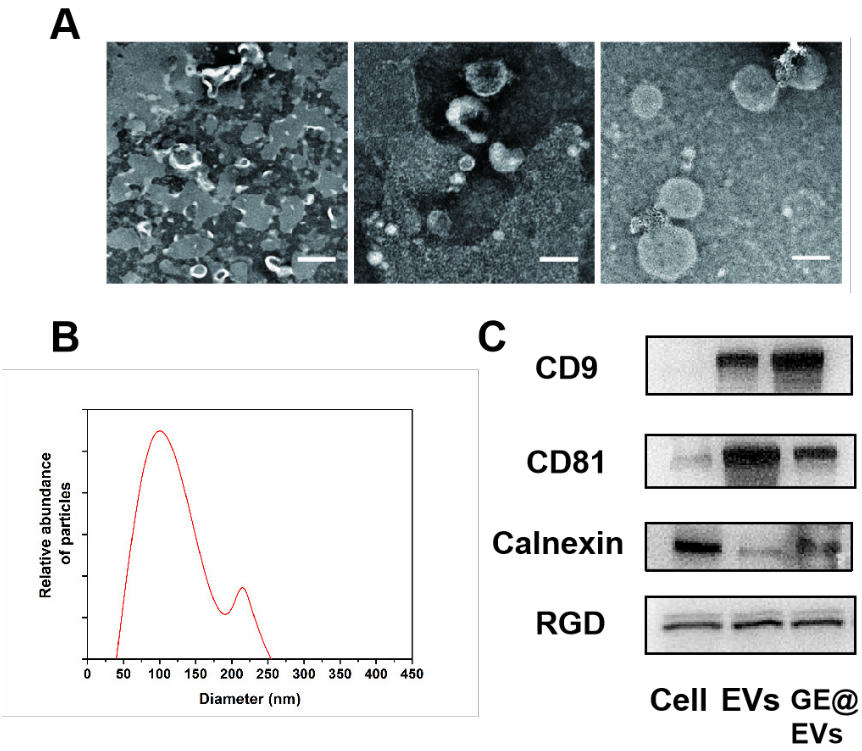

Figure S2. Characterization of EVs. (A) Electronic microscope observation of EVs. (scale bars: 200 nm, 100 nm, 50 nm). (B) Result of nanoparticle diameter analyses of the EVs. (C) EVs markers of CD9, CD81, Calnexin and RGD were analyzed by Western Blot.

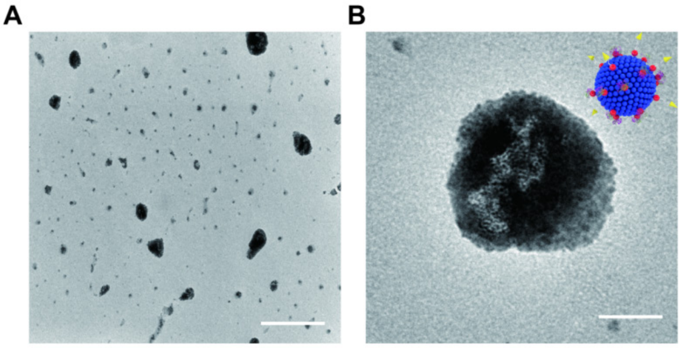

Figure S3. TEM images of the GE@EVs at (A) low and (B) high magnifications (scale bars: 500, 50 nm).

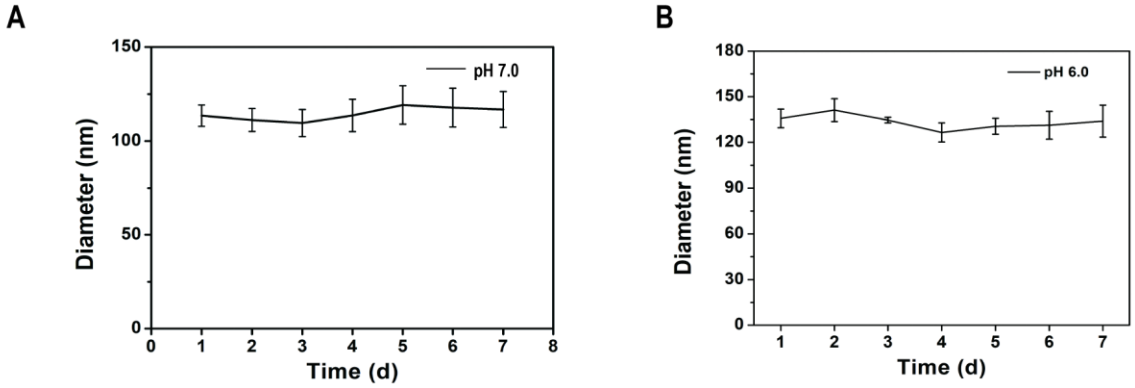

Figure S4. Stability experiment of GE@EVs at 4°C in PBS at pH (A) 7.0 and (B) 6.0 for 7 days.

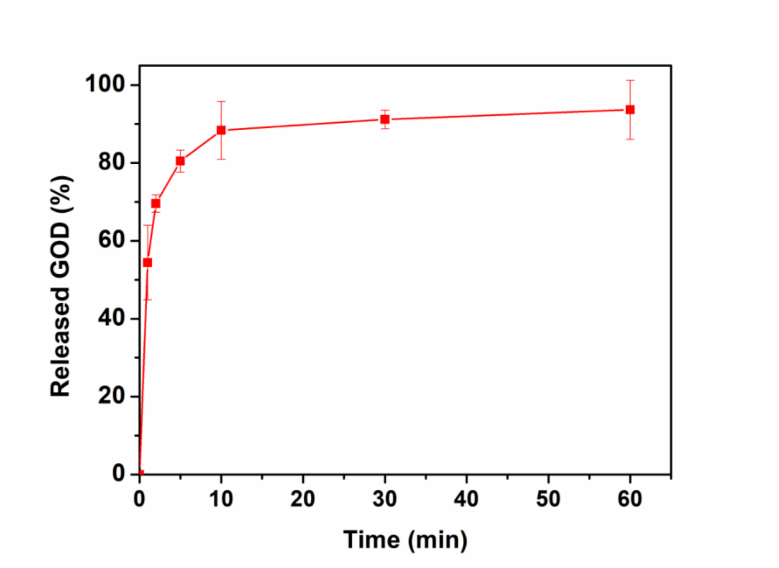

Figure S5. Cumulative release profiles of GOD in PBS using ultrasound to simulate the EVs breaking.

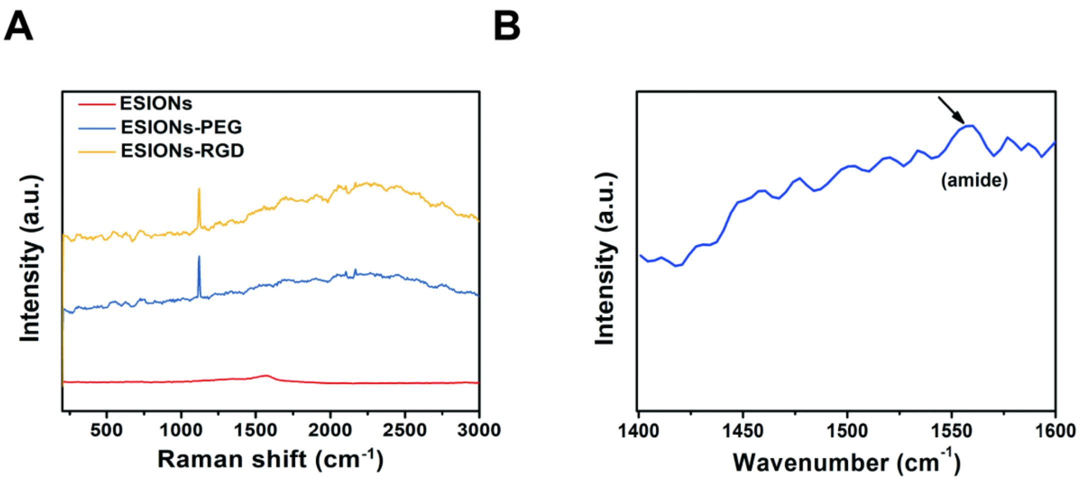

Figure S6. (A) Raman spectra of ESIONs, ESIONs-PEG, ESIONs-RGD. (B) Raman spectrum of ESIONs-RGD at 1400-1600 cm^{-1}.

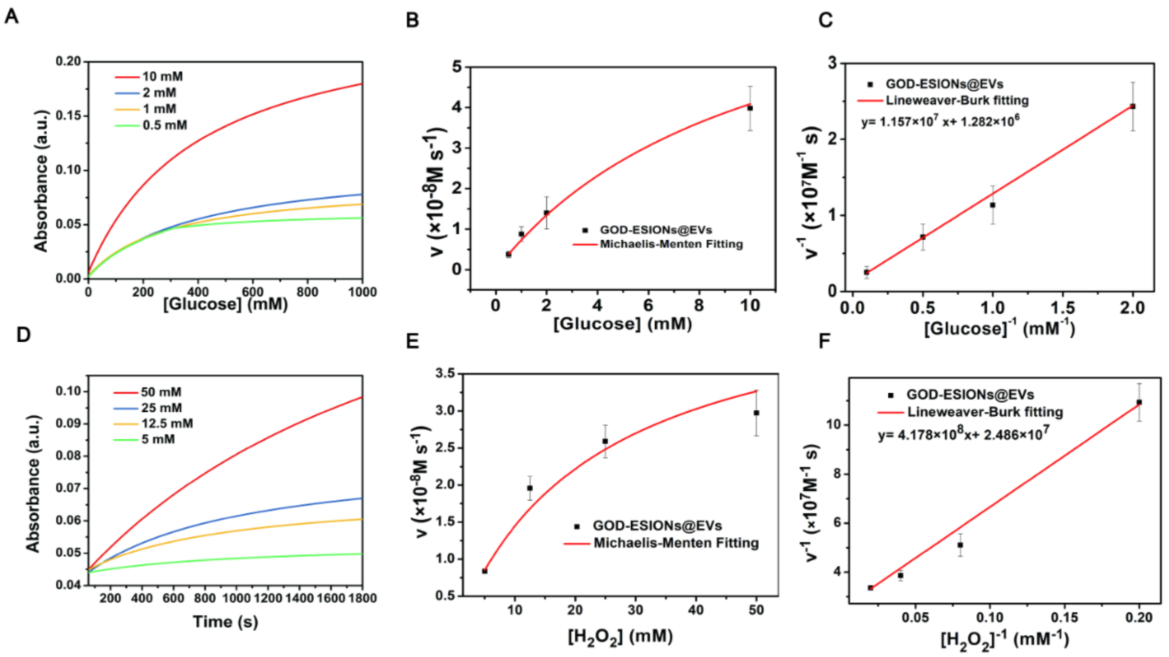

Figure S7. (A-F) Michaelis–Menten steady-state kinetics of GE@EVs in pH 7.4 solution. (A, D) Time-course absorbance of GE@EVs upon the addition of varied concentrations of β-D-glucose (10, 2, 1, and 0.5 mM) and H_2O_2 (50, 25, 12.5 and 5 mM). (B, C, E, F) Michaelis–Menten kinetics and Lineweaver–Burk plotting of GE@Exo with addition of β-D-glucose and H_2O_2.

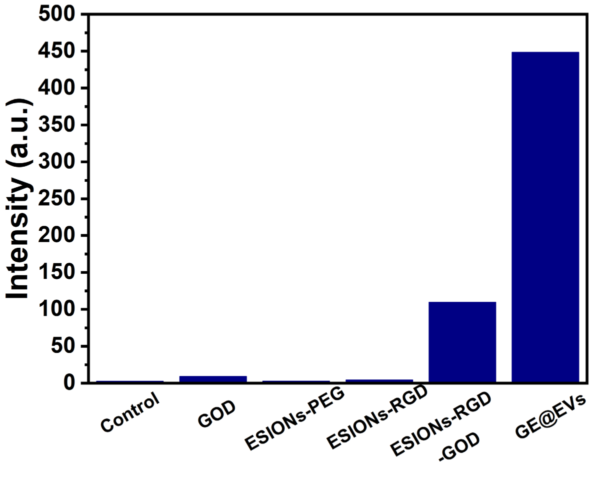

Figure S8. Fluorescent quantitation of huh 7 cells stained by DCFH-DA revealing the ROS production.

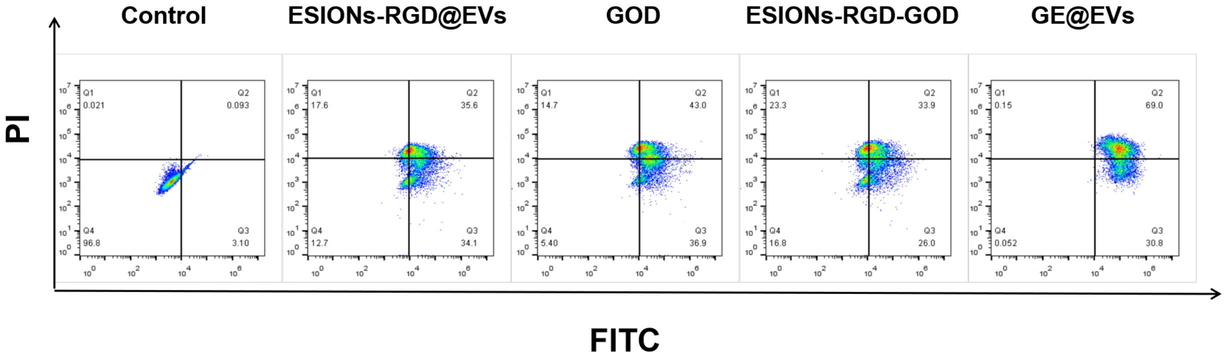

Figure S9. Flow cytometric analysis for evaluating the apoptosis and necrosis of Annexin V-FITC/PI-stained Huh 7 cells after different treatments for 12 h.

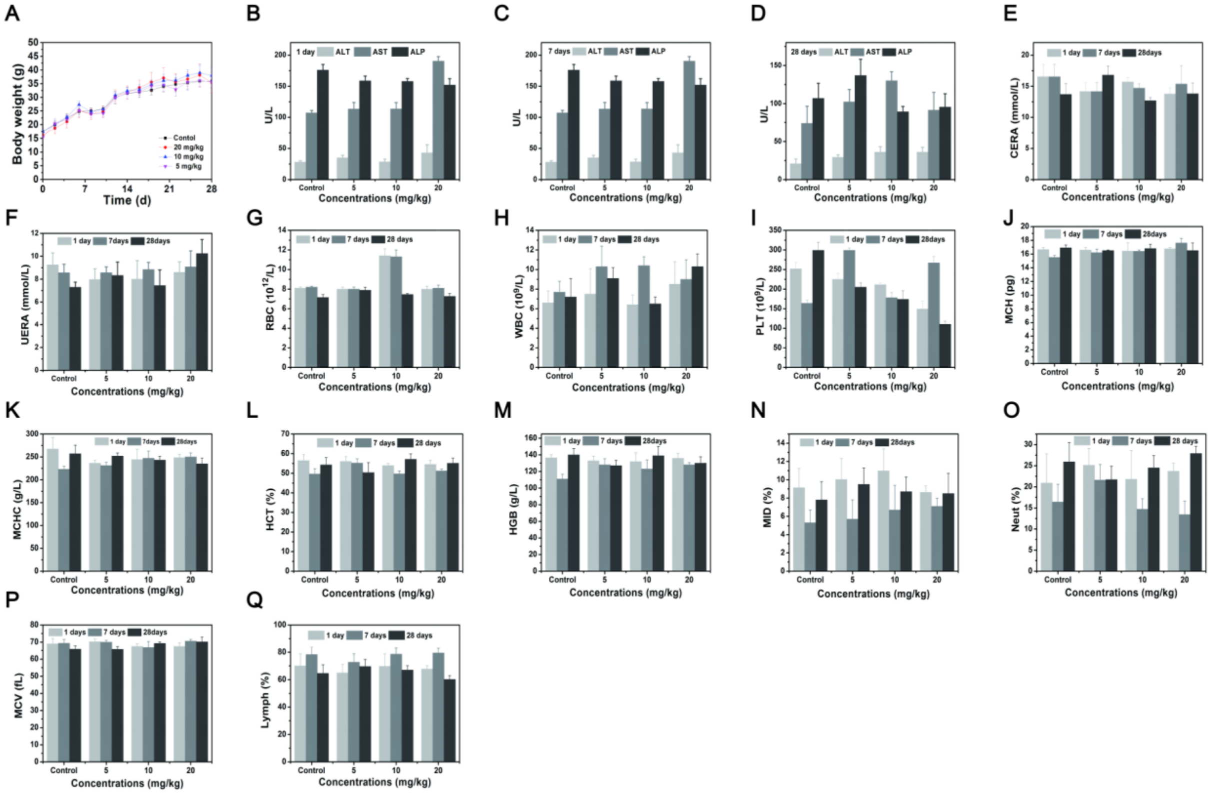

Figure S10. In vivo toxicity evaluation of ESIONs-RGD@EVs. (A) Time-dependent body-weight changing curves of Kunming mice during one-month feeding period. (B-F) Blood-biochemical analysis of the ESIONs-RGD@EVs treated mice in 1, 7 and 28 days injected with elevated doses (0, 5, 10, 20 mg/kg), including alanine aminotransferase (ALT), aspartate aminotransferase (AST), alkaline phosphatase (ALP), UREA, creatinine (CREA). (G-Q) Haematological index of the mice with intravenous administration of ESIONs-RGD@EVs (Fe doses: 0, 5, 10 and 20 mg/kg) in 1, 7 and 28 days. The results show the mean and s.d. values of red blood cell (RBC), white blood cell (WBC), platelet (PLT), mean corpuscular hemoglobin (MCH), mean corpuscular hemoglobin concentration (MCHC), mean corpuscular volume (MCV), hemoglobin (HGB), hematocrit (HCT), Median cell (MID), neutrophil (Neut), and lymphocyte (Lymph).

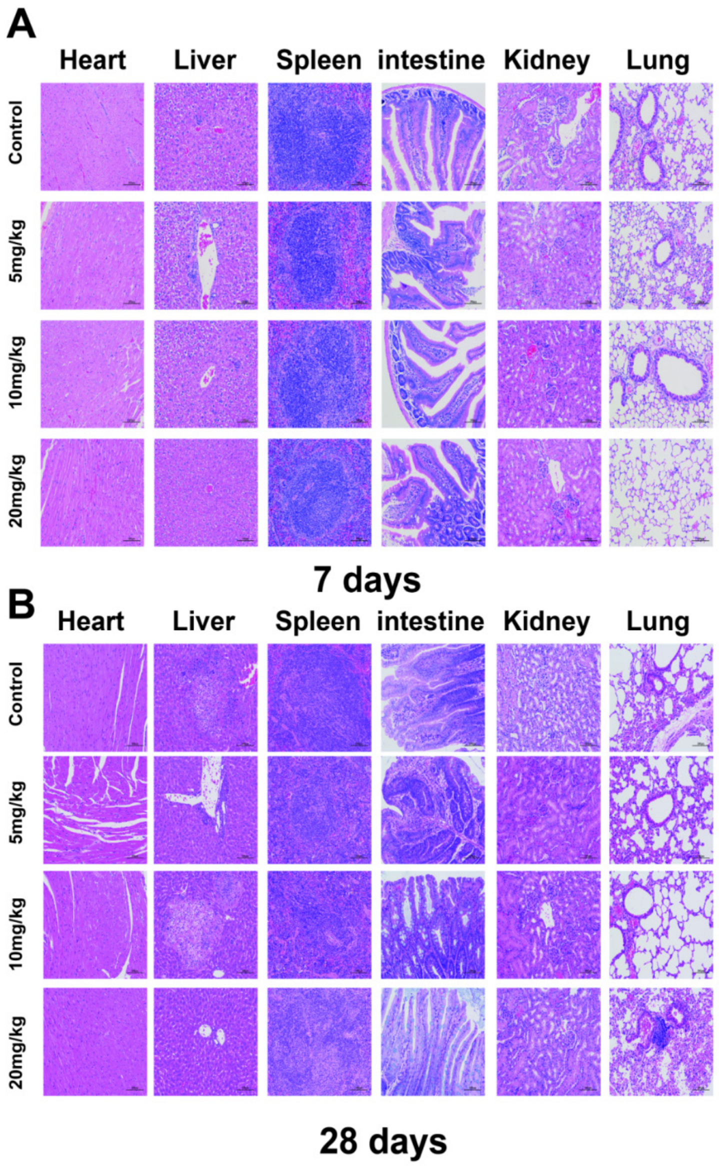

Figure S11. (A, B) Histological data (H&E stained images) obtained from the major organs (heart, liver, spleen, intestine, lung and kidney) of the ESIONs-RGD@EVs-treated mice at the 7th and 28th day. (scale bars: 100 µm).

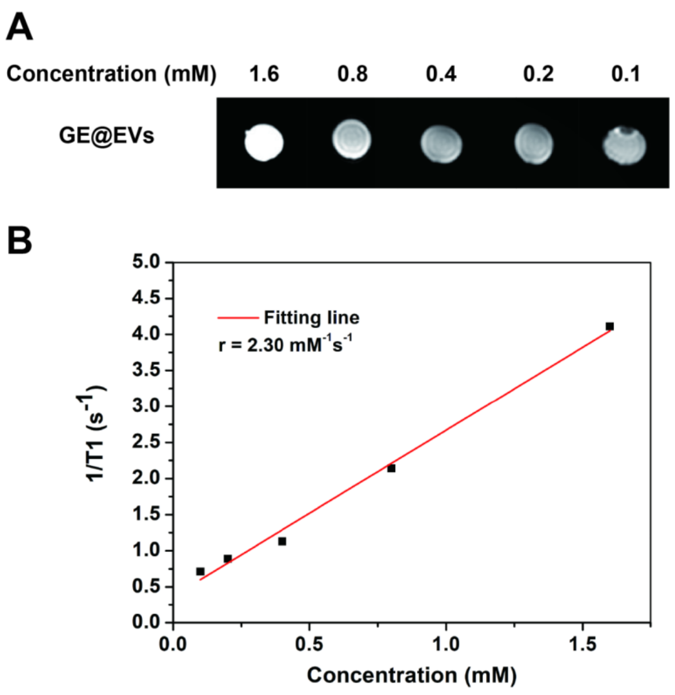

Figure S12. MR measurements of GE@EVs in vitro. (A) T_1-weighted MR images of GE@EVs at different concentrations. (B) Plots of GE@EVs against different concentrations.

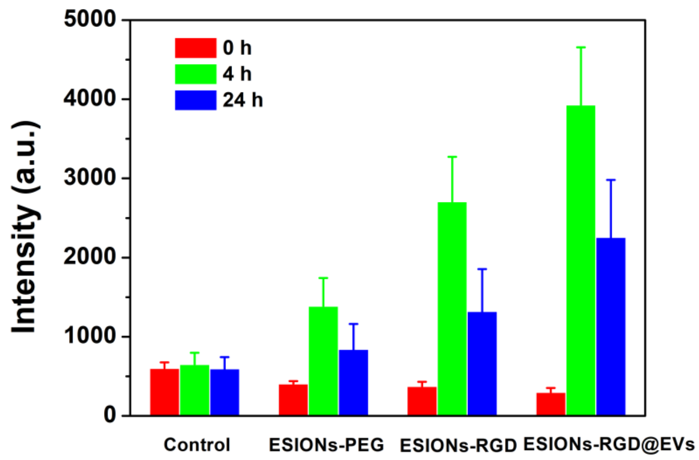

Figure S13. Fluorescent quantitation of Cy5.5-ESIONs-PEG, ESIONs-RGD and ESIONs-RGD@EVs *in vivo* at the tumor site.

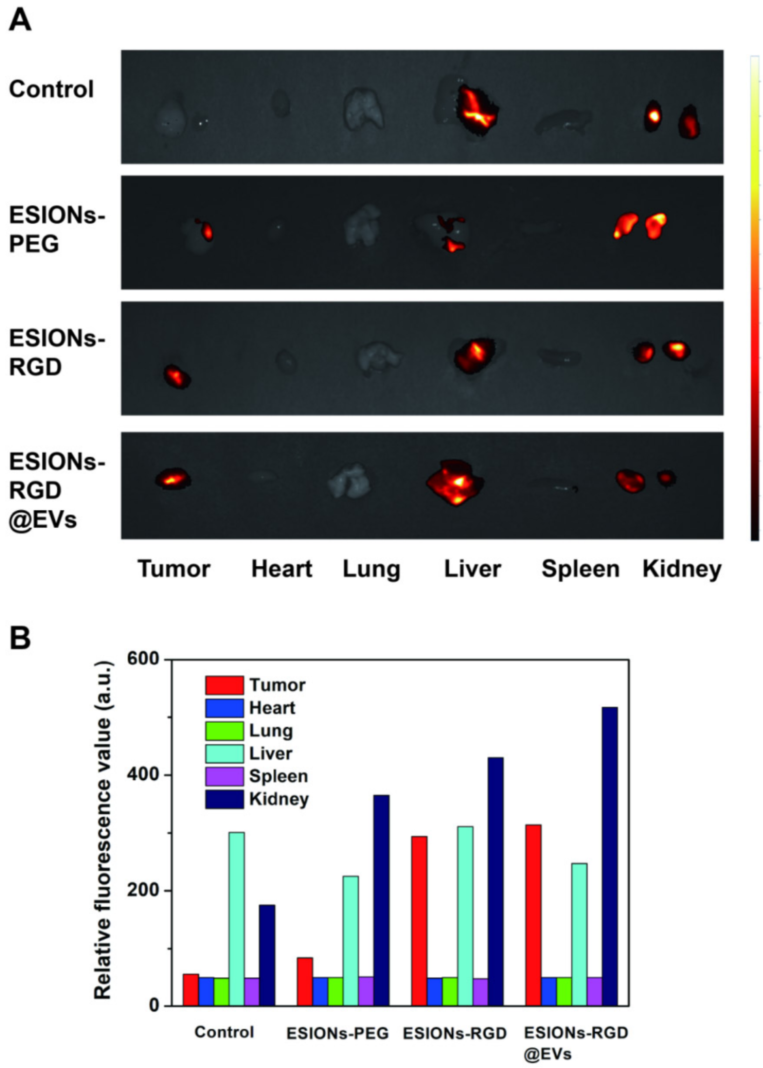

Figure S14. (A) Representative fluorescence images of major organs collected from mice at 4 hours after injection of Cy5.5-ESIONs-PEG, ESIONs-RGD and ESIONs-RGD@EVs. (B) Fluorescent quantitation of Cy5.5-ESIONs-PEG, ESIONs-RGD and ESIONs-RGD@EVs in different organs taken from the mice.
